# Potent Anticancer, Antimicrobial, and Anti‐Inflammatory Activities of *Boswellia sacra* Extracted Oil Nanoemulgel

**DOI:** 10.1155/bmri/7034598

**Published:** 2026-04-24

**Authors:** Ahmad M. Eid, Hani Naseef, Murad Abualhasan, Dana Yassin, Sally Jaber, Adan Abu Gazaleh

**Affiliations:** ^1^ Department of Pharmaceutical Chemistry and Technology, Faculty of Pharmacy, An-Najah National University, Nablus, State of Palestine, najah.edu; ^2^ Department of Cosmetic and Skin Care, Faculty of Pharmacy, An-Najah National University, Nablus, State of Palestine, najah.edu; ^3^ Pharmacy Department, Faculty of Pharmacy Nursing and Health Professions, Birzeit University, Birzeit, State of Palestine, birzeit.edu

**Keywords:** anticancer, anti-inflammatory, antimicrobial, *Boswellia sacra*, nanoemulgel, self-nanoemulsifying technique

## Abstract

**Introduction:**

*Boswellia sacra* (*B. sacra*), the chemical name for frankincense essential oil, is utilized extensively to treat multiple kinds of cancers as well as skin disorders like acne, infections, and inflammations. The goal of this project is to develop a nanoemulgel using *B. sacra oil* to enhance its antibacterial, anti‐inflammatory, and anticancer effects. *B. sacra* oil was extracted from oleoresin gum.

**Methods:**

The extracted oil was obtained via the steam distillation process. Then, nanoemulsion formulations were prepared using the self‐nanoemulsifying technique with Tween 80 and Span 80, incorporating the selected nanoemulsion formula. The resulting nanoemulsion was then incorporated with Carbopol hydrogel to produce nanoemulgel.

**Results:**

The nanoemulsion showed a droplet size of 120.03 nm and a PDI below 0.3. The zeta potential for nanoemulgel was −35 mV at 0.4% Carbopol concentration, and the nanoemulgel formulation demonstrated pseudo‐plastic behavior with no significant change in droplet size or PDI. The nanoemulgel has an antibacterial effect against various types of bacteria. The anticancer effect of *B. sacra* oil nanoemulgel was tested on different types of cancerous cells, which were: LX2, Hep‐G2, Hela, MCF‐7, and 3 T3, and the IC50 values were 186.2, 79.43, 186.2, 128.82, and 107.15 *μ*g/mL, respectively. The IC_50_ for COX‐1 was 4.3558 *μ*g/mL for *B. sacra* oil nanoemulgel and 1.3955 *μ*g/mL for COX‐2.

**Conclusion:**

The *B. sacra* oil nanoemulgel developed in this study has superior biological activities to those found in pure oil. Nanoemulgel is a promising delivery technique for improving several properties of pharmaceutical dosage forms.

## 1. Introduction

Since ancient times, medical plants have been utilized for therapeutic purposes, laying the foundation for a substantial portion of contemporary medicine. Modern drug manufacturers engage in comprehensive pharmacological screening of herbs as part of an ongoing effort to develop new medications [1]. Natural resources, including plants, animals, and minerals, offer products capable of treating a diverse array of human ailments. The preference for herbal therapy is on the rise compared with allopathic treatments, given the potential adverse effects associated with conventional medications. Patients favor natural remedies due to their perceived safety and lower incidence of side effects; approximately 80% of the general population turns to natural alternatives for various disorders [2]. Presently, herbs are employed in the treatment of both acute and chronic illnesses, spanning conditions such as inflammation, cardiovascular issues, prostate disorders, depression, and immune system support. Moreover, herbal products gain prominence when conventional medicine falls short in effectively addressing a condition, and they often complement other treatments, especially in cases of advanced cancer and emerging infectious diseases [3, 4].

In recent times, the extensive utilization of frankincense essential oil (scientifically known as *Boswellia sacra* [*B. sacra*]) for addressing skin concerns such as acne, infections, inflammations, and various types of cancer has been noted [5]. However, the scientific basis supporting these applications remains insufficient. This study, the first of its kind in Palestine, is aimed at address the widespread use of frankincense products on the skin without robust scientific backing for their efficacy in treating various conditions.

Frankincense, or olibanum, is an aromatic resin derived from the *Boswellia* tree genus, a member of the Burseraceae family [6]. Incisions in the trunk of the *Boswellia* tree yield an extruded resin, which, upon exposure to air, transforms into the recognizable orange–brown gum resin known as frankincense [7]. There are numerous *Boswellia* species, but our research focused on *B. sacra*, a species that is common in Arabic lands. *B. sacra* essential oil is produced by steam distillation of *B. sacra* resin. GC/MS analysis for hydrodistillate oil showed that the most abundant constituents were terpenes such as *α*‐pinene, *β*‐pinene, thujene, ocimene, camphene, limonene, sabinene, myrcene, sesquiterpenes, diterpenoids, triterpenoids, boswellic acids, and derivatives. These substances are also found in oil [8].

Numerous studies highlight the antimicrobial properties of *B. sacra* essential oil against fungi, especially *Candida albicans*, as well as gram‐negative and gram‐positive bacterial strains. Additionally, the essential oil exhibits antioxidant, immune‐modulating, and anti‐inflammatory effects [9]. Further investigations indicate its antiproliferative, proapoptotic, and cytotoxic effects on various cultured cancer cells, particularly breast cancer cells [6].

In recent years, nanotechnology has gained traction in pharmaceutics and drug delivery, offering innovative solutions for a range of applications [10–12]. Nanotechnology is a branch of science that specializes in materials at the nanoscale (1–1000 nm), based on the creation of nanostructures, which have revolutionary uses across several fields. Pharmaceutical nanotechnology enables us to create nanostructures that can improve novel drug delivery systems as therapeutic options for a range of illnesses [13]. Pharmaceutical nanotechnology enables the creation of nanostructures that enhance drug delivery systems, addressing challenges associated with the limited water solubility and bioavailability of pharmaceutical compounds. Nanocarriers, such as self‐nanoemulsifying drug delivery systems (SNEDDSs), show promise in overcoming these challenges by improving solubility, bioavailability, and modifying pharmacokinetics patterns [13, 14]. The bioavailability and water solubility of *B. sacra* essential oil are low. Thus, it is necessary to conduct research on techniques that can improve the solubility and bioavailability of the oil. For this, nanotechnology provides new strategies for modifying the physicochemical characteristics of phytochemical substances. Among these approaches are SNEDDSs [15].

SNEDDSs are compositions of surfactants, cosurfactants, and natural or synthetic oils that, with gentle agitation and dispersion in an aqueous phase like water, can form thermodynamically stable, transparent oil‐in‐water (O/W) nanoemulsions [16]. Typically, SNEDDSs yield nanoemulsions with droplet sizes ranging from 20 to 200 nm [17, 18]. Nanoemulsions offer various advantages, including enhanced drug solubility, high stability due to interfacial film formation, a transparent appearance, improved bioavailability, reduced systemic toxicity, and prevention of first‐pass metabolism. Despite these benefits, the low viscosity and spreadability of nanoemulsions can limit their topical use, potentially affecting patient compliance. This challenge has been addressed by transforming nanoemulsions into nanoemulgels [19]. Nanoemulgels are created by incorporating a nanoemulsion system into a hydrogel matrix, often using a gelling agent like Carbopol [18, 20]. In comparison to other topical dosage forms, nanoemulgels offer superior skin adherence, rheology, and spreadability. They are considered a practical choice for enhancing the topical administration of lipophilic medications, ensuring higher patient compliance, and minimizing toxic and irritant effects. Notably, nanoemulgels possess inherent high stability [18, 20]. This study explores the antimicrobial, anticancer, and anti‐inflammatory effects of *B. sacra* essential oil and a *B. sacra* oil‐nanoemulgel formulation.

## 2. Materials and Methods

### 2.1. Materials

Both surfactants (Span 80 and Tween 80) were sourced from Al‐Shams Company. We acquired Carboxyvinyl polymer (Carbopol 940) from CBC Co. Ltd., Japan. Dimethyl sulfoxide (DMSO) was sourced from Riedel De Haen, Germany. The Mueller Hinton agar, produced by Becton, Dickinson and Sparks Co. in France, was utilized in the culture of *B. sacra*. The *B. sacra* resin was obtained from Al‐Shams Company in Palestine. The plant resin underwent characterization in the Pharmacy Department at An‐Najah National University and is preserved under the voucher specimen code: Pharm‐PCT‐2806.

### 2.2. *B. sacra* Oil Extraction

After milling 500 g of *B. sacra*, 1500 mL of distilled water was placed in a well‐closed distillation flask. Next, this flask was exposed to a high temperature, close to 180°C. These contents were evaporated at the boiling point, and the vapor that condensed on the flask wall was then collected in a conical flask. The collected condensate was divided into two layers: a water layer at the bottom of the flask and the oil layer at the top. Finally, the oil layer was separated, and pure *B. sacra* oil was obtained [21]. One thousand, five hundred grams (about 3.31 lb) of *Boswellia* resin were used, and 6 g of *B. sacra* oil was extracted from these resins. Thus, the yield was 0.4%.

### 2.3. Preparation of *B. sacra* Resin Oil Nanoemulsion

For the goal of finding a ternary phase diagram, the oil was first extracted, and then it was turned into a nanoemulsion by adding a variety of combinations of surfactants (Tween 80 and Span 80) and *B. sacra* oil. This was done by a self‐emulsifying method. The amounts of these three ingredients varied between formulations. Each formulation was then gently mixed using the vortex for 3 min to homogenize it. The optimal formulation of *B. sacra* oil nanoemulsion was determined by evaluating the polydispersity index (PDI) and droplet size. Prior to assessing the *B. sacra* oil nanoemulsion formulation for droplet size, PDI, and physical characteristics, each formulation was self‐emulsified in distilled water through gentle agitation. A master size analyzer (Brookhaven Instruments, NanoBrook Omni, New York) was utilized to assess particle size and PDI [10, 22, 23]. The formulation composition, the detailed amounts and ratios of oil, surfactant, and cosurfactant are presented in the Supporting Information file.

### 2.4. Selection of *B. sacra* Oil Nanoemulsion Formulation

The optimum *B. sacra* oil nanoemulsion was selected based on the formulation that has the highest concentration *of B. sacra* oil with the smallest droplet size and least PDI.

### 2.5. Carbopol Hydrogel Formulation

Carbopol 940 was added to water, and the mixture was continually stirred until it became a homogenous mixture to prepare the hydrogel. Triethanolamine (TEA) was added to the mixture while stirring to adjust the pH of the hydrogel to 6. The Carbopol hydrogel was then allowed to rest for 24 h to complete gelation.

### 2.6. *B. sacra* Resin Oil Nanoemulgel Formulations

For preparing nanoemulgel formulations of *B. sacra* oil, first nanoemulsion formulations have been developed to find an optimum nanoemulsion of *B. sacra* oil using self‐nanoemulsifying technique, and then Carbopol hydrogel (which is the gelling agent) has been combined with the optimum nanoemulsion formulation. The self‐nanoemulsifying formulation that had already been optimized was first mixed with distilled water to make a nanoemulsion. Carbopol 940 was added to this nanoemulsion at three different levels (0.4%, 0.6%, and 0.8% *w*/*w*) to change it into a topical nanoemulgel. We mixed each combination very well until they were all uniform in appearance [23].

A master size analyzer (Brookhaven Instruments, United States) was used to measure the droplet size, PDI, and zeta potential of the nanoemulgel formulations. This method makes sure that the nanoscale properties of the nanoemulsion made from the self‐nanoemulsifying technique were still there after being mixed with the gel matrix.

### 2.7. Physical Characterization of *B. sacra* Oil Nanoemulgel Formulations

While we were in the process of producing the nanoemulgel, we visually evaluated a number of different physical qualities, such as its visual appearance, stability, permeability, homogeneity, and phase separation. A pH meter was used to monitor pH readings. (CG 820, Schott Gerate GmbH, Hofheim, Germany).

### 2.8. Analysis of *B. sacra* Oil Nanoemulgel Zeta Potential

The NanoBrook Omni was used to measure the zeta potential technique, which was applied as a parameter to detect the electrochemical equilibrium between nanoemulgel particles and liquid to estimate the stability and charge distribution of the particles. The zeta potential value was calculated as the average of three measurements, and it was then plotted against the concentration of Carbopol.

### 2.9. Rheological Characterization of *B. sacra* Oil Nanoemulgel

The rheological behavior of *B. sacra* oil nanoemulgel formulations that were prepared with different concentrations of Carbopol 940 (0.4, 0.6, and 0.8% Carbopol as a gelling agent) was tested using a spindle that was 7 s in size. A rheometer (Brookfield DVI, United States) with a shear rate range of 0–100 rpm was used in this experiment. The temperature was set at 25°C. Every measurement was performed three times. Then the sample′s density was multiplied by the obtained value to determine the viscosity [14].

### 2.10. Antimicrobial Activity of *B. sacra* Oil and Its Nanoemulgel

#### 2.10.1. Antibacterial

Methicillin‐resistant *Staphylococcus aureus* (MRSA), *Klebsiella pneumoniae* (*K. pneumoniae*) (ATCC 13883), *Pseudomonas aeruginosa* (*P. aeruginosa*) (ATCC 9027), *Staphylococcus aureus* (*S. aureus*) (ATCC 25923), *Proteus vulgaris* (*P. vulgaris*) (ATCC 90270), and *Escherichia coli* (*E. coli*) (ATCC 25922) were used to test the antibacterial activity of *B. sacra* oil and *B. sacra* oil nanoemulgel.

#### 2.10.2. Antifungal

*C. albicans* (ATCC 90028) was employed in the antifungal test.

#### 2.10.3. Culture Media

The culture medium, called Mueller Hinton agar, is made by mixing 17.5 g of acid hydrolysate of casein, 1.5 g of starch, 2 g of beef extract, and 17 g of agar per liter of purified water (manufactured by the French company Becton, Dickinson and Sparks Co.). Following a thorough blending of the ingredients, the mixture was simply heated to boiling in order to dissolve them. The combination was then held in the autoclave for 20 min at 121°C. Before being placed on sterile Petri plates, the agar was colored. We utilized a flat surface to provide a consistent surface and depth. The agar was then stored at 4°C–8°C.

The agar diffusion method was used to determine the antibacterial and antifungal activity. Agar‐filled plates were holed with four 6 mm‐diameter holes (A, B, C, and D) to complete this operation. Only DMSO was used to fill Hole A, whereas *B. sacra* oil and DMSO were used to fill Hole B, *B. sacra* nanoemulgel was used to fill Hole C, and Hole D was then filled with emulgel without using *B. sacra* oil as a guide. The antibacterial test required 24 h of incubation at 37°C on the plates. For the antifungal test, however, the plates were incubated for 24 h at 25°C. An essential step in determining the antibacterial and antifungal properties of a substance is the measurement of the inhibitory zone diameter [24].

### 2.11. Anticancer Effect of *B. sacra* Oil and Its Nanoemulgel

The study investigated the anticancer activity of *B. sacra* oil and *B. sacra* oil nanoemulgel across five distinct cancer cell lines compared with a positive control doxorubicin (DOX). A male hepatocellular carcinoma (HepG2; PRID: CVCL_0027) derived from liver cancer tissue, and human male hepatic stellate (LX‐2; PRID: CVCL_5792), which is a liver hepatic stellate cell; human female breast cancer (MCF‐7; PRID: CVCL_0031) and its derived from metastatic breast adenocarcinoma tissue; human female cervical adenocarcinoma (HeLa; PRID: CVCL_0030) derived from cervical adenocarcinoma; and a mouse embryo fibroblast cell line (3 T3; RRID:CVCL_0121) were maintained at 37°C in a humidified atmosphere with 5% CO_2_. The medium utilized was RPMI 1640 (Biological Industries, United Sates), supplemented with 10% fetal bovine serum, 1% penicillin/streptomycin, and 1% L‐glutamine. All cell lines were obtained from ATCC, Rockville, Maryland, United States on 1‐2025. To ensure the accuracy of the experimental results, ATCC used short tandem repeat (STR) profiling to authenticate each cell line and screened for mycoplasma contamination. Every experiment was carried out using aseptic techniques, and all cell culture work was carried out in a Class II Biosafety cabinet that had been previously cleaned with 70% alcohol in a water‐jacketed CO_2_ incubator.

The cells were distributed in 96‐well plates under suitable growth conditions, with approximately 10^3^ cells in a 100 *μ*L volume per well, in triplicate, and then incubated for a duration of 24 h. The culture medium was replaced with a new formulation that maintained the same base but included varying concentrations of *B. sacra* oil and *B. sacra* oil nanoemulgel (125, 250, 500, and 1000 *μ*g/mL), and the incubation duration was prolonged to 72 h. Additionally, following the guidelines provided by the manufacturer, the antiproliferative activity of the plant resin extracts was assessed using the CellTilter 96 Aqueous One Solution Cell Proliferation (MTS) Assay (Promega Corporation, Madison, Wsconsin). Following the procedure, each well received 20 *μ*L of the MTS solution per 100 *μ*L of medium, and this was subsequently incubated for 2 h at 37°C. Absorbance was measured at 490 nm [14].

### 2.12. Cyclooxygenase (COX) Analysis of *B. sacra* Oil and Its Nanoemulgel

Utilizing a COX inhibitor screening test kit (Item No: 560131), the capacity of *B. sacra* oil and its nanoemulgel to inhibit the conversion of arachidonic acid (AA) to PGH2 by bovine COX‐1 and human recombinant COX‐2 were assessed following the manufacturer′s instructions for the Cayman compound (United States). The experiment was performed on two occasions using two different concentrations (50 and 300 *μ*g/mL) to ascertain the drugs′ 50% inhibitory concentration (IC_50_) for COX‐1/COX‐2 activity. Following the guidelines provided in the kit instructions, a standard curve comprising eight concentrations of prostaglandin, along with samples for nonspecific binding and maximal binding, was utilized to assess the inhibition of the sample plant through the application of the multiple regression best‐fit line. The IC_50_ was established by analyzing the percentage inhibition at the two concentrations [25].

## 3. Results

### 3.1. *B. sacra* Oil Nanoemulsion Formulations Droplet Size and PDI Analysis

**Figure 1:**
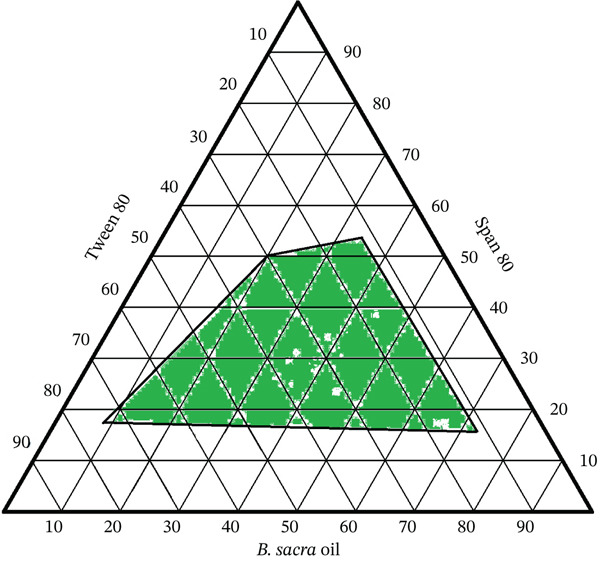
Pseudo ternary phase diagram of *B. sacra* oil nanoemulsion.

Through the use of a ternary phase diagram (Figure 1), several concentrations of the surfactants (Tween 80 and Span 80) and the oil from *B. sacra* were employed in order to determine the optimal formulation. The formulation composition and the detailed amounts and ratios of oil, surfactant, and cosurfactant have been provided as a Supporting Information. The optimal formulation for the nanoemulsion was selected on the basis of a PDI value that was lower than 0.3 and a droplet size that was smaller than 200 nm. Because of this, four different nanoemulsion formulations were selected for the purpose of comparison: 1, 2, 3, and 4. Formulation 1, which had a composition of 35% Tween 80, 15% Span 80, and 50% *B. sacra* oil, a PDI of 0.211, and a droplet size of 120.03 nm (Table 1), was the formulation that proved to be the most effective nanoemulsion formulation.

**Table 1:** The selected formulation of *B. sacra* oil nanoemulsion.

Formulation	Tween 80 (%)	Span 80 (%)	*B. sacra* oil (%)	Droplet size (nm)	Polydispersity index PDI
1	35	15	50	120.03 ± 5.07	0.211 ± 0.08
2	30	20	50	241.35 ± 4.53	0.272 ± 0.05
3	52	12	36	201.84 ± 6.81	0.279 ± 0.06
4	72	8	20	155.84 ± 4.90	0.211 ± 0.09

### 3.2. *B. sacra* Oil Nanoemulgel Formulations

Carbopol 940 was used as a gelling agent in order to accomplish the essential viscosity that was required for the formulation. The preparation of nanoemulgel was carried out using Carbopol 940 at several concentrations, namely 0.4%, 0.6%, and 0.8% by weight. First, the nanoemulsion was prepared through combining Tween 80 and Span 80 as mixed surfactants with *B. sacra* oil in distilled water; the nanoemulsion formulation was generated using the self‐emulsification process. After that, the Carbopol hydrogel was added while the mixture was continuously stirred in order to form the nanoemulgel. Several characteristics of the nanoemulgel formulation were analyzed, including its viscosity, droplet size, and size distribution.

### 3.3. Impact of Different Carbopol Concentrations on the PDI and Droplet Size of *B. sacra* Oil Nanoemulgel

**Figure 2:**
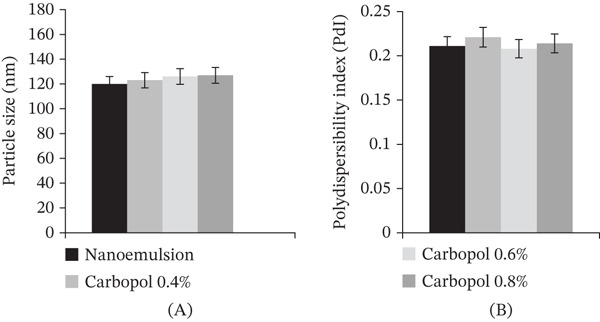
(A) The droplets size, (B) polydispersity index (PDI) of *B. sacra* oil nanoemulgel with different Carbopol concentrations.

For the purpose of determining the differences in PDI and droplet size, a comparison was done between the *B. sacra* oil optimum nanoemulsion and the *B. sacra* oil nanoemulgel that had different concentrations of Carbopol (0.4%, 0.6%, and 0.8%). When it comes to nanoemulgel formulations, the optimal formulation has the smallest droplet size and the lowest PDI (Figure 2).

### 3.4. *B. sacra* Oil Nanoemulgel Physical Characteristics

The selected *B. sacra* oil formulation demonstrates excellent spreadability, as we selected for the lowest concentration of Carbopol at 0.4%. The emulsification process occurs in under 30 s, resulting in a translucent appearance.

#### 3.4.1. Zeta Potential Measurement of *B. sacra* Oil Nanoemulgel

**Figure 3:**
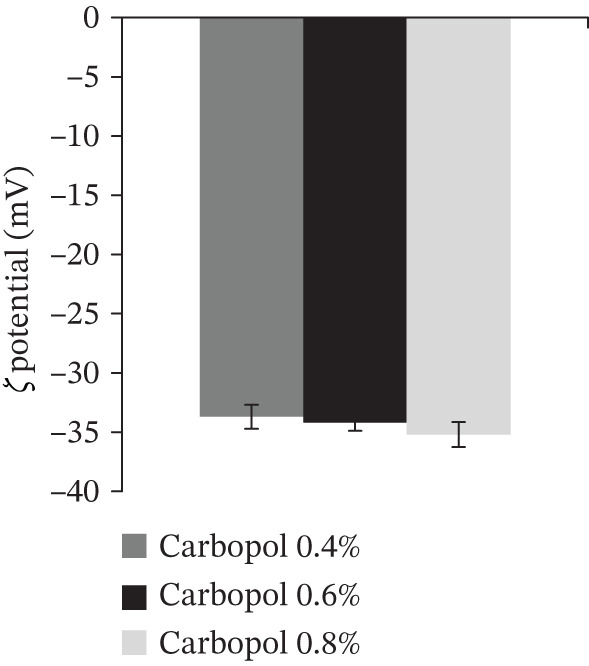
Zeta potential analysis of *B. sacra* oil nanoemulgel utilizing varying concentrations of Carbopol.

In accordance with the data shown in Figure 3, the zeta potential of every *B. sacra* oil nanoemulgel formulation was lower than −35.

#### 3.4.2. The Rheological Behavior of *B. sacra* Oil Nanoemulgel Formulations

**Figure 4:**
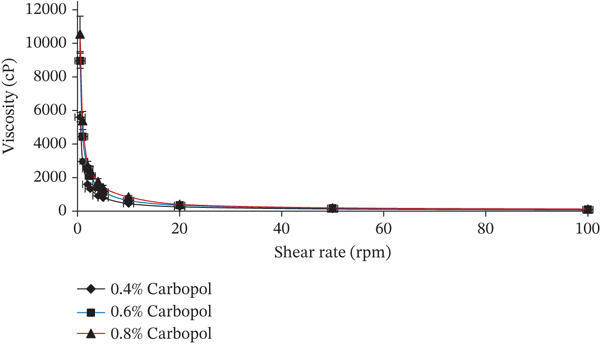
Investigation of the rheological properties of *B. sacra* oil nanoemulgel utilizing varying concentrations of Carbopol.

The deformation and flow behavior of a material are described and evaluated by its rheological behavior. These formulations exhibited pseudoplastic behavior because the viscosity decreased as the shear rate rose (Figure 4).

### 3.5. Antimicrobial Activity of *B. sacra* Oil and Its Nanoemulgel

The antibacterial activity of *B. sacra* oil and its nanoemulgel was tested on a variety of gram‐positive and gram‐negative bacteria. The research was conducted on both types of bacteria. Comparisons were made between the findings and those obtained from an antibiotic that served as a positive control and antifungal medications like ampicillin and fluconazole, respectively. In general, *B. sacra* oil and its nanoemulgel show antimicrobial activity in a wide range of bacteria, such as MRSA, *S. aureus*, *E. coli*, and *P. vulgaris*. On the other hand, both *B. sacra* oil and its nanoemulgel were not effective in inhibiting *K. pneumoniae* and *P. aeruginosa. B. sacra* oil nanoemulgel has a zone inhibition diameter of 22 mm on MRSA, which is near the zone inhibition diameter of the control‐positive ampicillin, which has a value of 26 mm on MRSA. *B. sacra* oil and its nanoemulgel show significant antimicrobial activity against *C. albican* with zone inhibition diameters of 24 and 29 mm, respectively, compared with the control‐positive drug fluconazole, which has a zone inhibition diameter of 12 mm (as shown in Table 2).

**Table 2:** Zone inhibition diameter (mm) of *B. sacra* oil and its nanoemulgel compared with ampicillin, ciprofloxacin, and fluconazole.

Microorganism	*B. sacra* oil	*B. sacra* oil nanoemulgel	Ampicillin	Fluconazole
MRSA	15 ± 0.8	22 ± 0.4	26 ± 1.4	—
*S. aureus* (ATCC 25923)	19 ± 0.3	28 ± 0.4	42 ± 0.7	—
*K. pneumonia* (ATCC 13883)	Resistant (no effect)	Resistant (no effect)	18 ± 1.4	—
*E. coli* (ATCC 25922)	11 ± 0.6	17 ± 0.4	33 ± 0.8	—
*P. aeruginosa* (ATCC 9027)	Resistant (no effect)	Resistant (no effect)	40 ± 0.7	—
*P. vulgaris* (ATCC 90270)	13 ± 0.3	22 ± 0.9	38 ± 2.8	—
*C. albican* (ATCC 90028)	24 ± 1.1	29 ± 0.6	—	12 ± 0.1

### 3.6. Anticancer Activity of *B. sacra* Oil and Its Nanoemulgel

**Figure 5:**
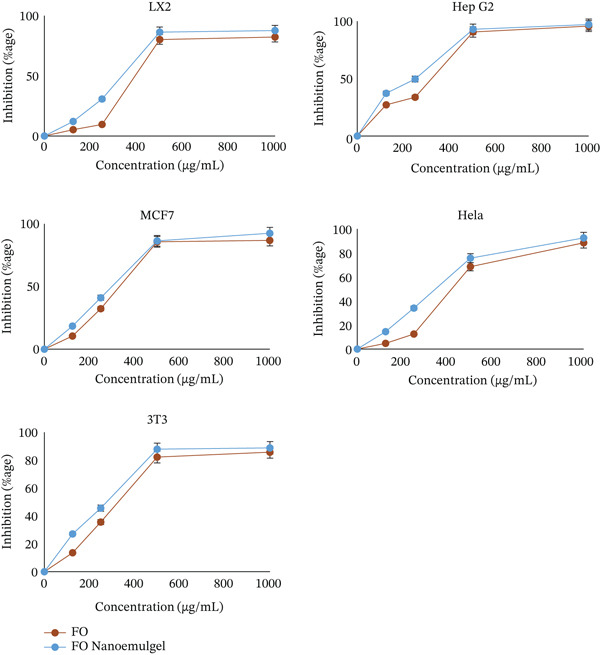
The cytotoxic effects of *B. sacra* oil (Frankincense oil [FO]) and its nanoemulgel.

The purpose of the study was to investigate the potential anticancer properties of *B. sacra* oil and its nanoemulgel by subjecting them to five distinct cell types: LX2, HepG2, Hela, MCF‐7, and 3 T3 cells. Following the completion of cytotoxic testing, we acquired some intriguing findings (Figure 5), which provide an explanation of the link between the concentration of *B. sacra* oil and its nanoemulgel, as shown by the percentage of cancer cell growth that was inhibited. As the concentration of oil and nanoemulgel grew, there was a corresponding rise in the suppression of the development of cancer cells. This indicates that oil and nanoemulgel work to limit the growth of cancer cells.

**Figure 6:**
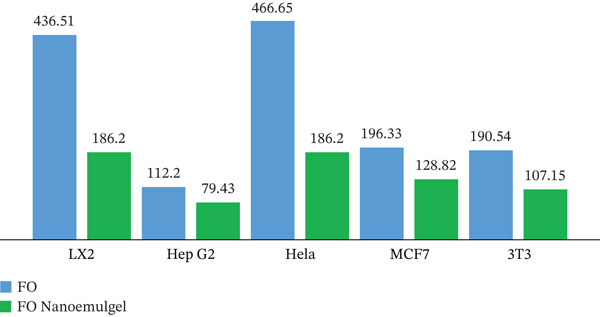
The values of the IC_50_ (*μ*g/mL) of *B. sacra* oil (Frankincense oil [FO]) and its nanoemulgel.

Figure 6 and Table 3 below illustrate the IC_50_ values of *B. sacra* oil and its nanoemulgel across various cell types. A decrease in IC_50_ corresponds to an increase in its effects on cancer cells. Hep‐G2 cells exhibit significant sensitivity to nanoemulgel and oil, showing an IC_50_ of 79.43*μ* and 112.2 *μ*g/mL, respectively. Nonetheless, Hela cells exhibited the lowest sensitivity to nanoemulgel and oil, presenting an IC_50_ of 186.2 *μ*g/mL and 466.65 *μ*g/mL, respectively. The impact of nanoemulgel and oil on LX2 cells was observed, yielding an IC_50_ of 186.2*μ* and 436.51 *μ*g/mL, respectively. MCF‐7 cells exhibited responses to nanoemulgel and oil, with IC_50_ values recorded at 128.82*μ* and 196.33 *μ*g/mL, respectively. The impact of nanoemulgel and oil on 3 T3 cells was observed, yielding IC_50_ values of 107.15*μ* and 190.54 *μ*g/mL, respectively, compared with the positive control DOX with an IC50 of 1.55 ± 1.35, 0.434 ± 0.271, 0.001 ± 0.0013, 0.05 ± 0.012 and 8.15 ± 2.09 *μ*g/mL for HeLa, Hep‐G2, MCF, LX‐2, and 3 T3, respectively. The nanoemulgel′s cytotoxic activity is more effective against cancer cells than the oil.

**Table 3:** The *B. sacra* oil and its nanoemulgel IC_50_ values (*μ*g/mL).

	LX2	Hep‐G2	Hela	MCF‐7	3 T3
*B. sacra* oil IC_50_ (*μ*g/mL)	436.51 ± 0.5	112.2 ± 0.3	466.65 ± 0.7	196.33 ± 0.9	190.54 ± 0.6
*B. sacra* oil nanoemulgel IC_50_ (*μ*g/mL)	186.2 ± 0.4	79.43 ± 0.8	186.2 ± 0.6	128.82 ± 0.7	107.15 ± 0.5

### 3.7. COX Inhibition Activity of *B. sacra* Oil and Its Nanoemulgel

**Figure 7:**
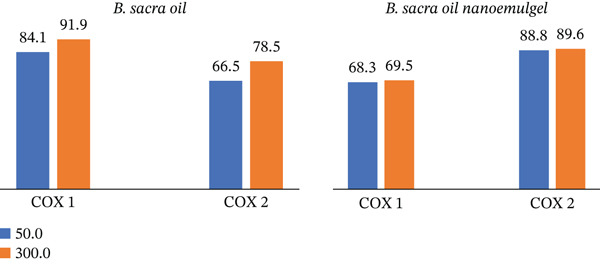
Percentage of inhibition activity of cyclooxygenase against COX‐1 and COX‐2 for *B. sacra* oil and its nanoemulgel.

**Figure 8:**
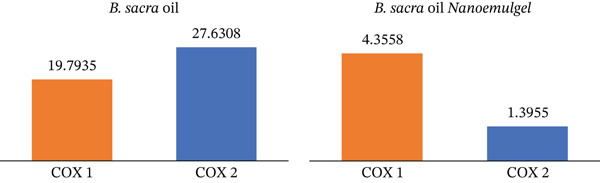
The IC_50_ values (*μ*g/mL) of *B. sacra* oil and its nanoemulgel.

Figure 7 shows that *B. sacra* oil and its nanoemulgel have COX inhibitory activity. *B. sacra* oil inhibits COX‐1 significantly more than COX‐2. This inhibition was improved when it was in the form of a nanoemulgel and was more selective for COX‐2. Figure 8 shows the IC_50_ values of *B. sacra* oil and its nanoemulgel. *B. sacra* oil has IC_50_ values of 19.7935 *μ*g/mL on COX‐1 and 27.6308 *μ*g/mL on COX‐2. As a result, *B. sacra* oil has anti‐inflammatory activity against both COX‐1 and COX‐2 but is more selective against COX‐1. The IC_50_ was improved when it was converted to nanoemulgel and became more selective for COX‐2, with an IC_50_ of 4.3558 *μ*g/mL on COX‐1 and 1.3955 *μ*g/mL on COX‐2.

## 4. Discussion

In this work, we investigated the enhancement of the therapeutic potential of *B. sacra* oil by developing a nanoemulgel. To do this, *B. sacra* oil was first prepared into a nanoemulsion using a self‐nanoemulsifying method and then mixed with Carbopol hydrogel. The constituents of the system, whether they are active or inactive, should be carefully selected in order to achieve an appropriate combination of oil, surfactant, and cosurfactant. This is necessary to produce a nanoemulsion using a self‐emulsification approach, which will have physiochemical characteristics acceptable for the final product [25].

The current study combines the extraction of *B. sacra* oil with its use in a SNEDDS‐based nanoemulsion, which then turns into a nanoemulgel. This lets us directly see how changes in the formulation affect the physicochemical attributes and biological performance. There have been reports of extracting *B. sacra* oil before, but the main improvement in this work is the logical design and optimization of the nano‐delivery system, not the extraction technique itself.

The optimized formulation (Table 1) had droplets smaller than 200 nm, which showed that the self‐emulsification process worked and that the chosen surfactant system was appropriate. This nanoscale range is very important since it is directly linked to a larger surface area and better dispersion stability, which makes it easier for biological targets to interact with them. The comparatively narrow size range and stability profile seen further point to good interfacial stabilization and resistance to coalescence over time. These results are in line with the reported increase in biological activity, which suggests that there is a direct link between nanoscale properties and functional performance [26].

A key factor in these results is the combined surfactant system made up of Tween 80 and Span 80. Span 80 was not a typical cosurfactant; instead, it was a secondary surfactant that changed the hydrophilic–lipophilic balance (HLB) and made the interfacial film more flexible. By using both high HLB (Tween 80) and low HLB (Span 80) surfactants, we were able to fine‐tune the effectiveness of emulsification, which led to better droplet size reduction and kinetic stability. This finding corroborates existing studies indicating that mixed surfactant systems offer enhanced interfacial packing and stabilization relative to single surfactants [14]. Only certain formulations with droplet sizes of 200 nm or below (Table 1) were moved on to further testing. This was done on purpose to improve the process, not by chance. The complete compositional variations included in the Supporting Information further illustrate that little alterations in surfactant ratios markedly affect droplet size and stability, highlighting the system′s sensitivity to formulation factors.

Transforming the optimized nanoemulsion into a nanoemulgel added another level of functional improvement. The higher viscosity and structured matrix should help the product stay on the skin longer and prevent runoff, which will allow for controlled release and longer residence time at the application site. This is especially important for dermal delivery systems because the stability and adherence of the formulation are two of the most important factors that affect how well it works.

The better performance seen in this study compared with earlier reports of essential oil‐based nanoformulations can be explained by the fact that multiple formulation variables were optimized at the same time. These variables include surfactant system design, droplet size control, and postemulsification gelation. The variability in previous findings is probably due to changes in the composition of the oil, the methods used to prepare it, and the fact that there was no systematic optimization. The current results underscore that nanoformulation performance is significantly formulation‐dependent, necessitating a systematic design strategy to attain reproducible and improved outcomes [27]. Rebolleda et al. investigated various surfactant types and showed that combined surfactants (Tween 80 and Span 80) had smaller droplet sizes than using a single surfactant. The outcomes also demonstrated that the O/W nanoemulsion of mixed surfactants (Tween 80 and Span 80) was stable [3, 27, 28]. Since it lowers droplet size and improves nanoemulsion stability, a range of HLB values between 10 and 12 is ideal for usage in O/W nanoemulsion. This is due to the hydrophilic nature of surfactants in that range, which favored the production and stability of O/W nanoemulsions [27].

It is necessary to develop a ternary phase diagram in order to determine the regions that are capable of self‐nanoemulsification and to choose the appropriate quantities of oil, surfactant, and cosurfactant that will be used in the formulation of the optimum SNEDDS. It is possible to build a formulation with a droplet size of less than 200 nm, and the diagrams represent the best formulation that could be developed [29].

The ability of SNEDDS to evaluate the amount and pace of drug release and absorption by measuring droplet size was an essential component that contributed to the success of the system. Furthermore, the increased interfacial surface area and the decreased droplet size led to an increase in the amount of biological activities that were carried out, which resulted in an even greater penetration. The SNEDDS criterion was satisfied when the droplet size was in the range of less than 200 nm. The appropriate mix of surfactant and cosurfactant resulted in a smaller globule size as well as a significant mechanical barrier that prevented the created globule from being aggregated, as was explained [30].

In the formulation of SNEDDS, the PDI, which is also known as the droplet size distribution to evaluate droplet homogeneity, is a factor considered as valuable as the droplet size. Once the PDI value is close to zero, the droplets are more homogeneous. Small values represent an emulsion with improved physical stability, homogeneity, and a narrower globule size distribution [3].

The PDI and droplet size of *B. sacra* oil nanoemulsion did not exhibit any significant changes when converted to nanoemulgel forms. The value of the zeta potential is used as a measure of the colloidal preparation′s stability. If the zeta potential of the droplets is virtually entirely negative or positive, then the droplets will reject one another, which will have the effect of maintaining the stability of the dispersion. When the values are low, dispersion becomes unstable because there is no energy to prevent the particles from clumping together. This is because there is no energy involved. It is common practice to establish a threshold of +30 or −30 mV as the border between stable and unstable dispersions [31]. Generally, the formulations were considered stable throughout when their zeta potential was either more than +30 mV or less than −30 mV. It is the presence of nonionic surfactants that causes the system to become more stable since they produce a coating on their surface. The stable nanoemulgel formulations exhibit a zeta potential value of approximately −35 mV [32].

For semisolid preparations, the rheological measures are crucial for describing the system physically, that is, flow characteristics, and controlling the stability, which is crucial for maintaining the efficacy and quality of the formulation. Viscosity is an important consideration since it can slow down drug diffusion out of the vehicle, which can alter drug release and bioavailability [33, 34]. The shear rate has dropped as the concentration of Carbopol has increased, and the viscosity has increased. The colloidal‐emulgel (nanoemulgel) demonstrated pseudoplastic rheological behavior, which means that viscosity decreased as shear rate increased. Because a substance′s viscosity affects its ability to diffuse through skin and its bioavailability, we selected the lowest concentration of Carbopol, which is 0.4%, to increase each of those properties [35].

Recently, the medical field has turned to using phytomedicines to treat microbial infections. In this research, we tested *B. sacra* oil nanoemulgel′s antibacterial efficacy. Comparing this nanoemulgel to *B. sacra* oil and the positive‐control antibiotic ampicillin, it shows a higher zone of inhibition. There are several causes for this bacterial suppression. First of all, because the particles are so tiny and have such a large surface area, the nanoemulgel penetrates the skin more deeply than oil or other drugs do. This boosts the nanoemulgel′s ability to contact bacteria [36].

Adebayo‐Tayo et al. had similar outcomes to our own, which demonstrated the effectiveness of a cream containing silver nanoparticles derived from *Senna alata* leaf extract in preventing bacterial and fungal development. They predicted that this cream′s improved penetration would result in an increase in the bacterial and fungal inhibition zones. However, the study revealed that the reason for the increased interaction between the bacteria and nanoemulgel was related to the packing process, which increases the amount of nanoemulgel that reaches the bacteria [37]. Furthermore, the use of *B. sacra* oil makes nanoemulgel more effective against both gram‐negative and gram‐positive bacteria. According to the Miran study published in 2022, *B. sacra* oil had an antibacterial effect on a variety of bacteria, including *P. aeruginosa*, *S. aureus*, and *Propionibacterium acnes* also exhibited a significant antifungal effect against *C. albicans* and *Malassezia furfur*. This effect was related to its composition of *α*‐pinene, *β*‐pinene, triterpenoids, and so on [8].

In this research, we discussed anticancer activities against the HeLa, MCF‐7, HepG2, LX2, and 3 T3 cell types. The particle size of our nanoemulgel (120.6 nm), which when viewed on a nanoscale implies a particle size of 100–200 nm, was a contributing factor to the impact of nanoemulgel that we observed. This size will enable its penetration into a tumor′s blood supply, producing cytotoxic effects. In 2012, Yue et al. found in their study that using PEGylated nanographene oxide as a nanocarrier increased the cytotoxic activity of graphene oxide because of its small particle size, which was ingested by the macrophage and then caused significant inflammation and the killing of cancer cells [38]. The presence of *B. sacra* oil induces proapoptotic, antiproliferative, and anti‐invasive properties, thereby reducing tumor aggressiveness in drug‐resistant and cultured metastatic tumors [39]. *B. sacra* oil was found to induce cancer cell death, prevent the formation of cellular networks in MDA‐MB‐231 cells on Matrigel, cause the breakdown of multicellular tumor spheroids (T47D cells), and regulate molecules involved in apoptosis, signal transduction, and cell cycle progression, according to the findings of a study that was conducted by Suhail et al. in particular, in regard to human breast cancer (MCF7) [6]. In 2022, Miran et al. confirmed the effectiveness of *B. sacra* oil in eradicating breast cancer, and he also discovered that it has efficacy on a pancreatic cancer cell line.in 2009 according to the findings of Pang et al., boswellic acid nanoparticles are responsible for the fragmentation of DNA that occurs during apoptosis. This fragmentation of DNA is the hallmark of apoptosis, which is caused by the nanoparticles when they are present [40].

Cyclooxygenase inhibition activity (COX‐1, COX‐2) was measured for *B. sacra* oil and *B. sacra* oil nanoemulgel. The majority of COX inhibitor drugs are taken orally; however, they have some side effects, such as irritation of the stomach lining, nausea, vomiting, and dizziness. Therefore, attention is drawn to using the nanoemulgel to inhibit COX due to its dermatological properties like being greaseless, easily spreadable, easily removable, emollient, nonstaining, pleasing in appearance, water‐soluble, having a longer shelf life, being bio‐friendly, and having high penetration, leading to better bioavailability. Khan et al. were successful in developing an aspirin emulgel formulation that improved the limitations of oral aspirin [41, 42]. Because of its chemical constituents such as pinene, ocimene, and bowsellic acid, *B. sacra* oil has an anti‐inflammatory effect as a COX‐1 and COX‐2 inhibitor [43]. However, the *B. sacra* oil nanoemulgel formulation showed an improvement in the anti‐inflammatory effect for both COX‐1 and COX‐2, with selectivity toward COX‐2, which means that *B. sacra* oil nanoemulgel has good anti‐inflammatory, analgesic, and antipyretic effects of NSAIDs, and at the same time, it has fewer side effects, which come from COX‐1 inhibition [44, 45]. Despite the promising results of the *B. sacra* nanoemulgel, a limitation of this study is the absence of cytotoxicity tests on normal human cell lines. Despite the formulation′s high efficacy, assessing its impact on healthy cells is necessary to calculate the selectivity index and confirm its biocompatibility. Future studies should include these safety tests to ensure the enhanced penetration and stability.

## 5. Conclusion

The nanoemulgel manufactured from *B. sacra* oil has shown a wide range of bioactive characteristics, including antibacterial, anti‐inflammatory, and anticancer activities, when compared with the qualities found in crude oil and the drug used as a positive control. When a nanoemulsion that contains *B. sacra* oil, Tween 80, and Span 80 is converted into a nanoemulgel by incorporating the hydrogel material Carbopol 940 at a concentration of 0.4%, the result is a formulation that has a smaller particle size and a narrower size distribution. This formulation allows for greater penetration through the skin. The rheological and physical characteristics of the nanoemulgel are exactly what would be expected. A promising step toward the use of simple nanotechnology methods in the formulation of pharmaceutical dosage forms will be represented by the findings that we achieved in this research.

**Nomenclature**
SNEDDSs: self‐nanoemulsifying drug delivery systems
O/W: oil‐in‐water
DMSO: dimethyl sulfoxide
PDI: polydispersity index
TEA: triethanolamine
ATCC: American Type Culture Collection
COX: cyclooxygenase
MRSA: methicillin‐resistant *Staphylococcus aureus*
IC_50_
: half‐maximal inhibitory concentration

## Author Contributions

**Ahmad M. Eid:** conceptualization, project administration, formal analysis, supervision, writing—original draft, writing—review and editing. **Hani Naseef:** investigation, methodology, project administration, writing—review and editing. **Murad Abualhasan:** formal analysis, writing—review and editing. **Dana Yassin:** writing—original draft, formal analysis. **Sally Jaber:** writing—original draft, formal analysis. **Adan Abu Gazaleh:** writing—original draft, formal analysis.

## Funding

No funding was received for this manuscript.

## Conflicts of Interest

The authors declare no conflicts of interest.

## Supporting information


**Supporting Information** Additional supporting information can be found online in the Supporting Information section. 

## Data Availability

The data that support the findings of this study are available from the corresponding authors upon reasonable request.

## References

[1] Dutra R. C. , Campos M. M. , Santos A. R. S. , and Calixto J. B. , Medicinal Plants in Brazil: Pharmacological Studies, Drug Discovery, Challenges and Perspectives, Pharmacological Research. (2016) 112, 4–29, 10.1016/J.PHRS.2016.01.021, 2-s2.0-84956633785, 26812486. 26812486

[2] Alara J. A. and Alara O. R. , Ethno-Medicinal Potentials and Phytochemical Properties of Aloe Vera: A Review, Journal of Chemical Engineering and Industrial Biotechnology. (2019) 5, 57–73, 10.15282/JCEIB.V5I1.3896.

[3] Eid A. M. , Istateyeh I. , Salhi N. , and Istateyeh T. , Antibacterial Activity of Fusidic Acid and Sodium Fusidate Nanoparticles Incorporated in Pine Oil Nanoemulgel, International Journal of Nanomedicine. (2019) 14, 9411–9421, 10.2147/IJN.S229557. 31819440 PMC6898994

[4] Naseef H. , Sahoury Y. , Farraj M. , Qurt M. , Abukhalil A. D. , Jaradat N. , Sabri I. , Rabba A. K. , and Sbeih M. , Novel Fusidic Acid Cream Containing Metal Ions and Natural Products Against Multidrug-Resistant Bacteria, Pharmaceuticals. (2022) 14, 10.3390/PHARMACEUTICS14081638. PMC9414967 36015264

[5] Kashyap N. , Kumari A. , Raina N. , Zakir F. , and Gupta M. , Prospects of Essential Oil Loaded Nanosystems for Skincare, Phytomedicine Plus. (2022) 2, 100198, 10.1016/J.PHYPLU.2021.100198.

[6] Suhail M. M. , Wu W. , Cao A. , Mondalek F. G. , Fung K. M. , Shih P. T. , Fang Y. T. , Woolley C. , Young G. , and Lin H. K. , *Boswellia sacra* Essential Oil Induces Tumor Cell-Specific Apoptosis and Suppresses Tumor Aggressiveness in Cultured Human Breast Cancer Cells, BMC Complementary and Alternative Medicine. (2011) 11, 1–14, 10.1186/1472-6882-11-129/FIGURES/7. 22171782 PMC3258268

[7] Van Vuuren S. F. , Kamatou G. P. P. , and Viljoen A. M. , Volatile Composition and Antimicrobial Activity of Twenty Commercial Frankincense Essential Oil Samples, South African Journal of Botany. (2010) 76, 686–691, 10.1016/J.SAJB.2010.06.001, 2-s2.0-78049245314.

[8] Miran M. , Amirshahrokhi K. , Ajanii Y. , Zadali R. , Rutter M. W. , Enayati A. , and Movahedzadeh F. , Taxonomical Investigation, Chemical Composition, Traditional Use in Medicine, and Pharmacological Activities of *Boswellia sacra* Flueck, Evidence-Based Complementary and Alternative Medicine. (2022) 2022, 8779676, 10.1155/2022/8779676. 35222678 PMC8881160

[9] Di Stefano V. , Schillaci D. , Cusimano M. G. , Rishan M. , and Rashan L. , In Vitro Antimicrobial Activity of Frankincense Oils From *Boswellia sacra* Grown in Different Locations of the Dhofar Region (Oman), Antibiotics. (2020) 9, 10.3390/ANTIBIOTICS9040195. PMC7235874 32325952

[10] Eid A. M. , Naseef H. , Jaradat N. , Ghanim L. , Moqadeh R. , and Yaseen M. , Antibacterial and Anti-Acne Activity of Benzoyl Peroxide Nanoparticles Incorporated in Lemongrass Oil Nanoemulgel, Gels. (2023) 9, 10.3390/GELS9030186. PMC10048723 36975635

[11] Aboofazeli R. , Nanometric-Scaled Emulsions (Nanoemulsions), Iranian Journal of Pharmaceutical Research. (2010) 94, 325–326, 10.22037/IJPR.2010.897. PMC3870055 24381596

[12] Al-Balushi R. A. , Haque A. , Saeed M. , Al-Harthy T. , Al-Hinaai M. , and Al-Hashmi S. , Unlocking the Anticancer Potential of Frankincense Essential Oils (FEOs) Through Nanotechnology: A Review, Molecular Biotechnology. (2024) 66, no. 11, 3013–3024, 10.1007/s12033-023-00918-5, 37914864. 37914864

[13] Douglas D. , Pharmaceutical Nanotechnology: A Therapeutic Revolution, International Journal of Pharmaceutical Sciences and Developmental Research. (2020) 6, 10.17352/IJPSDR.000027.

[14] Eid A. M. , Issa L. , Al-Kharouf O. , Jaber R. , and Hreash F. , Development of Coriandrum Sativum Oil Nanoemulgel and Evaluation of Its Antimicrobial and Anticancer Activity, BioMed Research International. (2021) 2021, 5247816, 10.1155/2021/5247816. 34671674 PMC8523232

[15] Nagaraja S. , Basavarajappa G. M. , Attimarad M. , and Pund S. , Topical Nanoemulgel for the Treatment of Skin Cancer: Proof-of-Technology, Pharmaceutical Journal. (2021) 13, 10.3390/PHARMACEUTICS13060902. PMC8234434 34207014

[16] Gurpreet K. and Singh S. K. , Review of Nanoemulsion Formulation and Characterization Techniques, Indian Journal of Pharmaceutical Sciences. (2018) 80, 781–789, 10.4172/PHARMACEUTICAL-SCIENCES.1000422.

[17] Shahavi M. H. , Hosseini M. , Jahanshahi M. , Meyer R. L. , and Darzi G. N. , Evaluation of Critical Parameters for Preparation of Stable Clove Oil Nanoemulsion, Arabian Journal of Chemistry. (2019) 12, 3225–3230, 10.1016/J.ARABJC.2015.08.024, 2-s2.0-84941007233.

[18] Eid A. M. , El-Enshasy H. A. , Aziz R. , and Elmarzugi N. A. , Preparation, Characterization and Anti-Inflammatory Activity of Swietenia Macrophylla Nanoemulgel, Journal of Nanomedicine and Nanotechnology. (2014) 5, 10.4172/2157-7439.1000190, 2-s2.0-84901999826.

[19] Choudhury H. , Gorain B. , Pandey M. , Chatterjee L. A. , Sengupta P. , Das A. , Molugulu N. , and Kesharwani P. , Recent Update on Nanoemulgel as Topical Drug Delivery System, Journal of Pharmaceutical Sciences. (2017) 106, 1736–1751, 10.1016/J.XPHS.2017.03.042, 2-s2.0-85019135257. 28412398

[20] Aman R. M. , Hashim I. I. A. , and Meshali M. M. , Novel Clove Essential Oil Nanoemulgel Tailored by Taguchi&rsquo;s Model and Scaffold-Based Nanofibers: Phytopharmaceuticals With Promising Potential as Cyclooxygenase-2 Inhibitors in External Inflammation, International Journal of Nanomedicine. (2020) 15, 2171–2195, 10.2147/IJN.S246601. 32280213 PMC7125334

[21] Said Al Amri A. , Jesil A. , and Salim A. , Extraction of Essential Oil From Frankincense Using Steam Distillation, International Journal of Trend in Research and Development. (2019) 6, 2394–9333, 10.1002/cjce.20.

[22] Eid A. M. , Naseef H. , Omar S. A. , Hawawerh A. , Baker Y. A. , Salameh J. , Sabri I. , and Zarour A. , Lavender-Oil Fusidic Acid Nanoemulgel: A Novel Strategy Against Fusidic Acid-Resistant *Staphylococcus aureus* , Nano Select. (2026) 7, e70134, 10.1002/nano.70134.

[23] Eid A. M. , Hawash M. , Abualhasan M. , Naser S. , Dwaikat M. , and Mansour M. , Exploring the Potent Anticancer, Antimicrobial, and Anti-Inflammatory Effects of Capparis Spinosa Oil Nanoemulgel, Coatings. (2023) 13, no. 8, 10.3390/COATINGS13081441.

[24] Dordević S. M. , Cekić N. D. , Savić M. M. , Isailović T. M. , Randelović D. V. , Marković B. D. , Savić S. R. , Stamenić T. T. , Daniels R. , and Savić S. D. , Parenteral Nanoemulsions as Promising Carriers for Brain Delivery of Risperidone: Design, Characterization and in Vivo Pharmacokinetic Evaluation, International Journal of Pharmaceutics. (2015) 493, 40–54, 10.1016/J.IJPHARM.2015.07.007, 2-s2.0-84938080952. 26209070

[25] Hawash M. , Jaradat N. , Hameedi S. , and Mousa A. , Design, Synthesis and Biological Evaluation of Novel Benzodioxole Derivatives as COX Inhibitors and Cytotoxic Agents, BMC Chemistry. (2020) 14, 1–9, 10.1186/S13065-020-00706-1/TABLES/1. 32944715 PMC7487730

[26] Jacob S. and Nair A. B. , Nanoemulgels as Advanced Topical Drug Delivery Systems: Mechanistic Insights and Therapeutic Applications in Skin Disorders, Infections, Wound Healing, and Cancer, Pharmaceutical Journal. (2026) 19, 10.3390/ph19020247. PMC12944252 41754788

[27] Chong W. T. , Tan C. P. , Cheah Y. K. , Lajis A. F. B. , Dian N. L. H. M. , Kanagaratnam S. , and Lai O. M. , Optimization of Process Parameters in Preparation of Tocotrienol-Rich Red Palm Oil-Based Nanoemulsion Stabilized by Tween80-Span 80 Using Response Surface Methodology, PLoS One. (2018) 13, e0202771, 10.1371/JOURNAL.PONE.0202771, 2-s2.0-85052191911. 30142164 PMC6108518

[28] Rebolleda S. , Sanz M. T. , Benito J. M. , Beltrán S. , Escudero I. , and González San-José M. L. , Formulation and Characterisation of Wheat Bran Oil-in-Water Nanoemulsions, Food Chemistry. (2015) 167, 16–23, 10.1016/J.FOODCHEM.2014.06.097, 2-s2.0-84904277918. 25148953

[29] Mayer S. , Weiss J. , and McClements D. J. , Vitamin E-Enriched Nanoemulsions Formed by Emulsion Phase Inversion: Factors Influencing Droplet Size and Stability, Journal of Colloid and Interface Science. (2013) 402, 122–130, 10.1016/J.JCIS.2013.04.016, 2-s2.0-84878170910. 23660020

[30] Nepal P. R. , Han H. K. , and Choi H. K. , Preparation and In Vitro–In Vivo Evaluation of Witepsol H35 Based Self-Nanoemulsifying Drug Delivery Systems (SNEDDS) of Coenzyme Q10, European Journal of Pharmaceutical Sciences. (2010) 39, 224–232, 10.1016/J.EJPS.2009.12.004, 2-s2.0-75149137336. 20035865

[31] Arriaga L. R. , Drenckhan W. , Salonen A. , Rodrigues J. A. , Íñiguez-Palomares R. , Rio E. , and Langevin D. , On the Long-Term Stability of Foams Stabilised by Mixtures of Nano-Particles and Oppositely Charged Short Chain Surfactants, Soft Matter. (2012) 8, 11085–11097, 10.1039/C2SM26461G, 2-s2.0-84867648995.

[32] Wahgiman N. A. , Salim N. , and Abdul Rahman M. B. , Phase Behaviour Study on Medium-Chain Triglyceride/Surfactant/Water Systems Containing Gemcitabine Using Phase Inversion Composition Technique, Malaysian Journal of Analytical Sciences. (2020) 24, 363–372.

[33] Boddupalli B. M. , Mohammed Z. N. K. , Nath A. R. , and Banji D. , Mucoadhesive Drug Delivery System: An Overview, Journal of Advanced Pharmaceutical Technology and Research. (2010) 1, 381–387, 10.4103/0110-5558.76436, 2-s2.0-79952979545. 22247877 PMC3255397

[34] Ma′ali A. , Naseef H. , Qurt M. , Abukhalil A. D. , Rabba A. K. , and Sabri I. , The Preparation and Evaluation of Cyanocobalamin Mucoadhesive Sublingual Tablets, Pharmaceuticals. (2023) 16, 10.3390/PH16101412/S1. PMC10610133 37895883

[35] Sahu S. K. and Sharma A. K. , Influence of Carbopol and Polyox on the Release of Metoprolol Succinate From Extended-Release Matrix Tablets: A Doe Approach and In-Vivo Evaluation, World Journal of Pharmacy and Pharmaceutical Sciences. (2018) 7, 10.20959/wjpps20184-11343.

[36] Arbain N. H. , Salim N. , Wui W. T. , Basri M. , and Rahman M. B. A. , Optimization of Quercetin Loaded Palm Oil Ester Based Nanoemulsion Formulation for Pulmonary Delivery, Journal of Oleo Science. (2018) 67, 933–940, 10.5650/JOS.ESS17253, 2-s2.0-85051081277. 30012897

[37] Adebayo-Tayo B. C. , Borode S. O. , and Alao S. O. , In–Vitro Antibacterial and Antifungal Efficacy of Greenly Fabricated *Senna alata* Leaf Extract Silver Nanoparticles and Silver Nanoparticle-Cream Blend, Periodica Polytechnica Chemical Engineering. (2022) 66, 248–260, 10.3311/PPCH.18271.

[38] Pei X. , Zhu Z. , Gan Z. , Chen J. , Zhang X. , Cheng X. , Wan Q. , and Wang J. , PEGylated Nano-Graphene Oxide as a Nanocarrier for Delivering Mixed Anticancer Drugs to Improve Anticancer Activity, Scientific Reports. (2020) 10, no. 1, 10.1038/s41598-020-59624-w, 32066812. PMC7026168 32066812

[39] Parsonidis P. , Vlachou I. , Mamagkaki A. , Bouris I. , Daikopoulou V. , and Papasotiriou I. , Cytotoxic Effect of *Boswellia sacra* on Human Cancer Cell, Journal of Cancer Science and Therapy. (2021) 13, 1–1, 10.37421/1948-5956.2021.13.487.

[40] Pang X. , Yi Z. , Zhang X. , Sung B. , Qu W. , Lian X. , Aggarwal B. B. , and Liu M. , Acetyl-11-Keto-*β*-Boswellic Acid Inhibits Prostate Tumor Growth by Suppressing Vascular Endothelial Growth Factor Receptor 2-Mediated Angiogenesis, Cancer Research. (2009) 69, 5893–5900, 10.1158/0008-5472.CAN-09-0755/655269/P/ACETYL-11-KETO-BOSWELLIC-ACID-INHIBITS-PROSTATE. 19567671 PMC2724674

[41] Khan J. , Norfarhani S. , Sahu R. K. , Ruhi S. , Kaleemullah M. , Al-Dhalli S. , Rasny M. , Budiasih S. , Baber S. , Ng C. H. , Shamiha N. N. , Khan G. M. , Gamal O. E. , Fattepur S. , Nilugal K. , Abdullah I. , Asmani F. , and Yusuf E. , Development and Evaluation of Topical Emulgel of Aspirin Using Different Polymeric Bases, Research Journal of Pharmacy and Technology. (2020) 13, no. 12, 6300–6304, 10.5958/0974-360X.2020.01096.3.

[42] Al-Balushi R. A. , Chaudhuri A. , Kandimalla R. , Haque A. , Alenezi K. M. , Saeed M. , Changez M. , Al Harthy T. , Al Hinaai M. , Siddiqui S. , Agrawal A. K. , and Aqil F. , In Vitro Anticancer Effects of Frankincense and Its Nanoemulsions for Enhanced Cancer Cell Targeting, Frontiers in Pharmacology. (2025) 16, 1403780, 10.3389/fphar.2025.1403780. 39981177 PMC11839425

[43] Li X. J. , Yang Y. J. , Li Y. S. , Zhang W. K. , and Tang H. , Bin *α*-Pinene, Linalool, and 1-Octanol Contribute to the Topical Anti-Inflammatory and Analgesic Activities of Frankincense by Inhibiting COX-2, Journal of Ethnopharmacology. (2016) 179, 22–26, 10.1016/J.JEP.2015.12.039, 2-s2.0-84951763357. 26721216

[44] Famaey J. P. , In Vitro and In Vivo Pharmacological Evidence of Selective Cyclooxygenase-2 Inhibition by Nimesulide: An Overview, Inflammation Research. (1997) 46, 437–446, 10.1007/S000110050221/METRICS. 9427063

[45] Naseef H. , Al-Maharik N. , Rabba A. K. , Sharifi-Rad M. , Hawash M. , Jaradat N. , Naseef H. , Al-Maharik N. , Rabba A. K. , Sharifi-Rad M. , Hawash M. , and Jaradat N. , Phytochemical Characterization and Assessments of Antimicrobial, Cytotoxic and Anti-Inflammatory Properties of Lavandula Coronopifolia Poir. Volatile Oil from Palestine, Arabian Journal of Chemistry. (2022) 15, 104069, 10.1016/j.arabjc.2022.104069.

