# Supplementary material for: Potent Anticancer, Antimicrobial, and Anti‐Inflammatory Activities of Boswellia sacra Extracted Oil Nanoemulgel
Source: Biomed Res Int. 2026 Apr 24;2026:7034598. doi: 10.1155/bmri/7034598 (PMC13108431; doi:10.1155/bmri/7034598)
Supplement: Supplementary file 1 — Supporting Information Additional supporting information can be found online in the Supporting Information section. [file BMRI-2026-7034598-s001.pdf]

Potent Anticancer, Antimicrobial, and Anti-inflammatory Activities of *Boswellia Sacra* Extracted Oil Nanoemulgel

Formulation composition, the detailed amounts and ratios of oil, surfactant, and co-surfactant

AUTOMATIC PSEUDO DIAGRAM OF NANOEMULSIFYING SYSTEMS

Formulation 190

Number	Surfactant A (%)	Surfactant B (%)	Oil (%)
1	10	90	0
2	8	72	20
3	6.4	57.6	36
4	5.12	46.08	48.8
5	4.096	36.86	59.04
6	3.277	29.49	67.23
7	2.621	23.59	73.79
8	2.097	18.87	79.03
9	1.678	15.1	83.22
10	1.342	12.08	86.58
11	1.074	9.664	89.26
12	0.859	7.731	91.41
13	0.687	6.185	93.13
14	0.55	4.948	94.5

Formulation 280

Number	Surfactant A (%)	Surfactant B (%)	Oil (%)
1	20	80	0
2	16	64	20
3	12.8	51.2	36
4	10.24	40.96	48.8
5	8.192	32.77	59.04
6	6.554	26.21	67.23
7	5.243	20.97	73.79
8	4.194	16.78	79.03
9	3.355	13.42	83.22
10	2.684	10.74	86.58
11	2.147	8.59	89.26
12	1.718	6.872	91.41
13	1.374	5.498	93.13
14	1.1	4.398	94.5

Formulation 370

Number	Surfactant A (%)	Surfactant B (%)	Oil (%)
1	30	70	0
2	24	56	20
3	19.2	44.8	36
4	15.36	35.84	48.8
5	12.29	28.67	59.04
6	9.83	22.94	67.23
7	7.864	18.35	73.79
8	6.291	14.68	79.03
9	5.033	11.74	83.22
10	4.027	9.395	86.58
11	3.221	7.516	89.26
12	2.577	6.013	91.41
13	2.062	4.81	93.13
14	1.649	3.848	94.5

Formulation 460

Number	Surfactant A (%)	Surfactant B (%)	Oil (%)
1	40	60	0
2	32	48	20
3	25.6	38.4	36
4	20.48	30.72	48.8
5	16.38	24.58	59.04
6	13.11	19.66	67.23
7	10.49	15.73	73.79
8	8.389	12.58	79.03
9	6.711	10.07	83.22
10	5.369	8.053	86.58
11	4.295	6.442	89.26
12	3.436	5.154	91.41
13	2.749	4.123	93.13
14	2.199	3.299	94.5

Formulation 550

Number	Surfactant A (%)	Surfactant B (%)	Oil (%)
1	50	50	0
2	40	40	20
3	32	32	36
4	25.6	25.6	48.8

5	20.48	20.48	59.04
6	16.38	16.38	67.23
7	13.11	13.11	73.79
8	10.49	10.49	79.03
9	8.389	8.389	83.22
10	6.711	6.711	86.58
11	5.369	5.369	89.26
12	4.295	4.295	91.41
13	3.436	3.436	93.13
14	2.749	2.749	94.5

Formulation 640

Number	Surfactant A (%)	Surfactant B (%)	Oil (%)
1	60	40	0
2	48	32	20
3	38.4	25.6	36
4	30.72	20.4	48.8
5	24.58	16.38	59.04
6	19.66	13.11	67.23
7	15.73	10.49	73.79
8	12.58	8.389	79.03
9	10.07	6.711	83.22
10	8.053	5.369	86.58
11	6.442	4.295	89.26
12	5.154	3.436	91.41
13	4.123	2.749	93.13
14	3.299	2.199	94.5

Formulation 730

Number	Surfactant A (%)	Surfactant B (%)	Oil (%)
1	70	30	0
2	56	24	20
3	44.8	19.2	36
4	35.84	15.36	48.8
5	28.67	12.29	59.04
6	22.94	9.83	67.23
7	18.35	7.864	73.79
8	14.68	6.294	79.03
9	11.74	5.033	83.22
10	9.395	4.027	86.58
11	7.516	3.221	89.26
12	6.013	2.577	91.41
13	4.81	2.062	93.13
14	3.848	1.649	94.5

Formulation 820

Number	Surfactant A (%)	Surfactant B (%)	Oil (%)
1	80	20	0

2	64	16	20
3	51.2	12.8	36
4	40.96	10.24	48.8
5	32.77	8.192	59.04
6	26.21	6.554	67.23
7	20.97	5.243	73.79
8	16.78	4.194	79.03
9	13.42	3.355	83.22
10	10.74	2.684	86.58
11	8.59	2.147	89.26
12	6.872	1.718	91.41
13	5.498	1.374	93.13
14	4.398	1.1	94.5

Formulation 910

Number	Surfactant A (%)	Surfactant B (%)	Oil (%)
1	90	10	0
2	72	8	20
3	57.6	6.4	36
4	46.08	5.12	48.8
5	36.86	4.096	59.04
6	29.49	3.277	67.23
7	23.59	2.621	73.79
8	18.87	2.097	79.03
9	15.1	1.678	83.22
10	12.08	1.342	86.58
11	9.664	1.074	89.26
12	7.731	0.859	91.41
13	6.185	0.687	93.13
14	4.978	0.55	94.5
